# Supplementary material for: Approach of Pregnant Women from Poland and the Ukraine to COVID-19 Vaccination—The Role of Medical Consultation
Source: Vaccines (Basel). 2022 Feb 8;10(2):255. doi: 10.3390/vaccines10020255 (PMC8880363; doi:10.3390/vaccines10020255)
Supplement: Supplementary file 1 [file vaccines-10-00255-s001.zip › vaccines-1549065-supplementary.pdf]

Suplementary Materials

Table S1. General characteristics.

	**Total N = 300**	**Poland N = 150**	**Ukraine N=150**	***P*-value**
Age, years	25.2 ± 4.3	25.5 ± 4.5	24.9 ± 4.1	0.16
Arterial hypertension	20 (6.7)	10 (6.7)	10 (6.7)	1.0
Epilepsy	9 (3)	4 (2.7)	5 (3.3)	1.0
Diabetes mellitus	32 (10.7)	16 (10.7)	16 (10.7)	1.0
Heart defect	8 (2.7)	3 (2)	5 (3.3)	0.72
Bronchial asthma	14 (4.7)	4 (2.7)	10 (6.7)	0.1
Pancreatitis	10 (3.3)	6 (4)	4 (2.7)	0.52
Obesity	31 (10.3)	11 (7.3)	20 (13.3)	0.08
Psoriasis	11 (3.7)	11 (7.3)	0 (0)	<0.001
Migraine	5 (1.7)	2 (1.3)	3 (2)	1.0
Thrombophilia	10 (3.3)	4 (2.7)	6 (4)	0.52
Celiac disease	8 (2.7)	5 (3.3)	3 (2)	0.72
Sclerosis multiplex	9 (3)	7 (4.7)	2 (1.33)	0.17
Allergy	17 (5.7)	3 (2)	14 (9.3)	0.006
Depression	9 (3)	6 (4)	3 (2)	0.5
Bipolar affective disease	5 (1.7)	3 (2)	1 (1.3)	1.0
Melanoma	2 (0.7)	1 (0.7)	1 (0.7)	1.0

Cerebral tumour	1 (0.7)	0 (0)	1 (0.7)	1.0
Hypothyreosis	22 (7.3)	9 (6)	13 (8.7)	0.37
Data are presented as mean ± SD and counts (percentages).				

Table S2. Socioeconomic indices.

	**Total** N = 300	**Poland** N = 150	**Ukraine** N=150	***P*-value**
**Place of residence**				
Rural area	49 (16.3)	16 (10.7)	33 (22)	0.09
City < 50,000 residents	78 (26)	33 (22)	45 (30)	
City < 250,000 residents	137 (45.7)	93 (62)	44 (29.3)	
City ≥ 250,000 residents	36 (12)	8 (5.3)	28 (18.7)	
Education				
Primary	37 (12.3)	14 (9.3)	23 (15.3)	0.03
Vocational	53 (17.7)	23 (15.3)	30 (20)	
Secondary	108 (36)	56 (37.3)	52 (34.7)	
Higher	102 (34)	57 (38)	45 (30)	
Prior births	180 (60)	89 (59.3)	91 (60.7)	0.57

Number of prior births	1.14 ± 0.98	1.01 ± 0.93	1.28 ± 1.02	0.02
Number of children	1.09 ± 0.97	0.99 ± 0.9	1.2 ± 1.03	0.1
Prior miscarriages	0.74 ± 0.75	0.75 ± 0.77	0.73 ± 0.73	0.91
Currently in relationship				0.8
Married	190 (63.3)	94 (62.7)	96 (64)	
Partnership	94 (31.3)	49 (32.7)	45 (30)	
No relationship	16 (5.3)	7 (4.7)	9 (6)	
Trimester of pregnancy:				0.8
first	46 (15.3)	23 (15.3)	23 (15.3)	
second	144 (48)	76 (50.7)	68 (45.3)	
third	110 (36.7)	51 (34)	59 (39.3)	
Chronic diseases	140 (46.7)	70 (46.7)	70 (46.7)	1.0
Medications	137 (45.7)	72 (48)	65 (43.3)	0.41
Allergies	97 (32.3)	46 (30.7)	51 (34)	0.07
Has your doctor talked to you about the COVID-19 vaccination?	84 (28.1)	45 (30.2)	39 (26)	0.59
Do you think that the course of COVID-19 disease in pregnancy, compared to non-pregnant women, is usually:				0.32

milder	145 (48.6)	69 (46.3)	76 (51)	
comparable	61 (20.5)	31 (20.8)	30 (20.1)	
more dangerous	61 (20.5)	32 (21.5)	29 (19.5)	
much more dangerous	31 (10.4)	17 (11.4)	14 (9.4)	
Do you think that immunity obtained by disease lasts longer than immunity acquired by vaccination?				<0.001
definitely yes	110 (36.7)	49 (32.7)	61 (40.7)	
probably yes	87 (29)	35 (23.3)	52 (34.6)	
probably not	58 (19.3)	33 (22)	25 (16.7)	
definitely not	45 (15)	33 (23)	12 (8)	
What do you think is the effectiveness of COVID-19 vaccines in protecting against the disease (what percentage of people vaccinated will not contract COVID-19)?				<0.001
About 20%	77 (25.7)	21 (14)	56 (37.4)	
About 40%	94 (31.3)	50 (33.3)	44 (29.3)	
About 70%	67 (22.3)	34 (22.7)	33 (22.0)	
Above 90%	62 (20.7)	45 (30)	17 (11.3)	

Do you think that vaccination against COVID-19 is safe in pregnancy?				<0.001
Yes	78 (26)	54 (36)	24 (16)	
No	128 (42.7)	55 (36.7)	73 (48.7)	
It depends on the trimester of pregnancy	47 (15.7)	27 (18)	20 (13.3)	
I do not know	47 (15.7)	14 (9.3)	33 (22)	
Do you think the complications after receiving the COVID-19 vaccine are:				<0.001
very rare	27 (9)	18 (12)	9 (6)	
rare	89 (29.7)	54 (36)	35 (23.3)	
do not know	22 (7.3)	6 (4)	16 (10.7)	
common	88 (29.3)	44 (29.3)	44 (29.3)	
very common	74 (24.7)	28 (18.7)	46 (30.7)	
Do you think that the complications of undergoing COVID-19 vaccine in pregnancy, compared to non-pregnant women are:				0.02
less dangerous	29 (9.7)	11 (7.3)	18 (12)	
equally dangerous	83 (27.7)	53 (35.4)	30 (20)	

more dangerous	151 (50.3)	69 (46)	82 (54.7)	
do not know	37 (12.3)	17 (11.3)	20 (13.3)	
Is the COVID-19 vaccine dangerous to the foetus ?				0.02
ys	164 (54.7)	78 (52)	86 (57.3)	
no	56 (18.7)	37 (24.7)	19 (12.7)	
do not know	60 (20)	29 (19.3)	29 (21.7)	
insufficient research on this topic	20 (6.6)	6 (4)	14 (9.3)	
Have you been vaccinated against COVID-19?				0.002
yes	11 (3.7)	7 (4.7)	4 (2.7)	
yes, before pregnancy	16 (5.3)	14 (9.3)	2 (1.3)	
yes, during pregnancy	5 (1.7)	4 (2.7)	1 (0.7)	
first and second dose during pregnancy,	6 (2)	4 (2.7)	2 (1.3)	
I postponed the first dose before pregnancy - full vaccination until later in pregnancy	12 (4)	8 (5.3)	4 (2.7)	
I postponed the first dose during pregnancy - full vaccination after pregnancy	15 (5)	11 (7.3)	4 (2.7)	
no	235 (78.3)	102 (68)	133 (88.7)	

In the previous question, the answer was 'no'. Reason for the negative approach:				
1) Fear of post-vaccination complications/adverse reactions	173 (57.7)	65 (43.3)	108 (72)	<0.001
2) Fear of damage to the foetus	201 (67)	102 (68)	99 (66)	0.71
3) Fear of complications for pregnancy such as pre-term delivery	103 (34.3)	30 (20)	73 (48.7)	<0.001
4) I believe that vaccinations are ineffective	55 (18.3)	4 (2.7)	51 (34)	<0.001
5) I believe that disease is better than vaccination and provides more long-lasting immunity	17 (5.7)	2 (1.3)	15 (10)	0.001
6) I have not had such an opportunity yet due to limitations in the vaccination programme	12 (4)	2 (1.3)	10 (6.7)	0.02
7) Other reasons	3 (1)	1 (0.7)	2 (1.3)	1.0
Are you concerned about the COVID-19 vaccination during pregnancy?				<0.001
yes	114 (38.3)	41 (27.5)	73 (49)	
probably yes	103 (34.6)	55 (36.9)	48 (32.2)	
probably no	60 (20.1)	35 (23.5)	25 (16.8)	

definitely not	21 (7)	18 (12.1)	3 (2)	
Are you going to be vaccinated against COVID-19 when you are pregnant (if you have not yet been vaccinated)?				<0.001
yes	37 (12.3)	27 (18)	10 (6.7)	
probably yes	41 (13.7)	26 (17.3)	15 (10)	
do not know	45 (15)	25 (16.7)	20 (13.3)	
no	140 (46.7)	56 (37.3)	84 (56)	
definitely not	37 (12.3)	16 (10.7)	21 (14)	
The question concerns women who answered 'no'/'rather not'/'definitely not' to the previous question. Reason for the negative approach:				
1) Fear of post-vaccination complications/adverse reactions	189 (63)	85 (56.7)	104 (69.3)	0.02
2) Fear of damage to the foetus	178 (59.3)	78 (52)	100 (66.7)	0.01
3) Fear of complications for pregnancy such as pre-term delivery	110 (36.7)	41 (27.3)	69 (46)	<0.001
4) I believe that vaccinations are ineffective	46 (15.3)	8 (5.3)	38 (25.3)	<0.001

5) I believe that disease is better than vaccination and provides more long-lasting immunity	9 (3)	2 (1.3)	7 (4.7)	0.17
6) Other reasons	1 (0.3)	0 (0)	1 (0.7)	1.0
Have you vaccinated your children against COVID-19?				0.002
yes	33 (11)	23 (15.3)	10 (6.6)	
no	122 (40.7)	47 (31.3)	74 (50)	
I have not had such an opportunity yet due to the age restrictions in the vaccination programme	114 (38)	68 (45.4)	46 (30.7)	
I do not have children yet	29 (10.3)	11 (8)	18 (12.7)	
The question concerns women who answered 'no' in the previous question. Reason for the negative approach:				
1) Risk of post-vaccination complications/undesirable effects	187 (62.3)	93 (62)	94 (62.7)	0.9
2) I believe that vaccinations are ineffective	87 (29)	27 (18)	60 (40)	<0.001
3) I believe that disease is better than vaccination and provides more long-lasting immunity	65 (21.7)	22 (14.7)	43 (28.7)	0.003
4) Other reasons	23 (7.7)	6 (4)	17 (11.3)	0.02

Are you planning to vaccinate your children against COVID-19?				<0.001
yes	46 (15.4)	31 (20.7)	15 (10.1)	
probably yes	49 (16.4)	28 (18.7)	21 (14.1)	
do not know	82 (27.4)	40 (26.7)	42 (28.2)	
no	106 (35.4)	47 (31.3)	59 (39.6)	
definitely not	16 (5.3)	4 (2.7)	12 (8)	
The question concerns women who answered 'no'/'rather not'/'definitely not' to the previous question. Reason for the negative approach:				
1) Risk of post-vaccination complications/undesirable effects	184 (61.3)	88 (58.7)	96 (64)	0.34
2) I believe that vaccinations are ineffective	94 (31.3)	35 (23.3)	59 (39.3)	0.003
3) I believe that disease is better than vaccination and provides more long-lasting immunity	73 (24.3)	26 (17.3)	47 (31.3)	0.005
4) Other reasons:	28 (9.3)	8 (5.33)	20 (13.3)	0.02
Would you like your relatives to get vaccinated against COVID-19?				0.04

yes	64 (21.3)	39 (26)	25 (16.7)	
yes, but only relatives at risk of severe disease	66 (22)	33 (22)	33 (22)	
do not know	68 (22.7)	33 (22)	35 (23.3)	
no	93 (31)	42 (28)	51 (34)	
definitely not	9 (3)	3 (2)	6 (4)	
The question concerns women who answered 'no'/'rather not'/'definitely not' to the previous question. Reason for the negative approach:				
1) Risk of post-vaccination complications/undesirable effects	187 (62.3)	88 (58.7)	99 (66)	0.19
2) I believe that vaccinations are ineffective	95 (31.7)	34 (22.7)	61 (40.7)	<0.001
3) I believe that disease is better than vaccination and provides more long-lasting immunity	56 (18.7)	17 (11.3)	39 (26)	0.001
4) Other reasons	17 (5.7)	4 (2.7)	13 (8.7)	0.02

Table S3. Other questions.

Has your doctor talked to you about the COVID-19 vaccination?	**Total**<br>**N = 300**	**Poland**<br>**N = 150**	**Ukraine**<br>**N=150**	***P*-value**
Do you think that the course of COVID-19 disease in pregnancy, compared to non-pregnant women, is usually:				
milder	13 (4.3)	5 (3.3)	8 (5.4)	
comparable	62 (20.7)	38 (25.3)	24 (16.1)	
more dangerous	103 (34.4)	31 (20.7)	72 (48.3)	
much more dangerous	121 (40.5)	76 (50.7)	45 (30.2)	
Do you think that immunity obtained by disease lasts longer than immunity acquired by vaccination?				<0.001
definitely yes	25 (8.4)	7 (4.7)	18 (12.1)	
probably yes	50 (16.8)	14 (9.5)	36 (24.2)	
probably not	83 (27.9)	30 (20.3)	53 (35.6)	
definitely not	139 (46.8)	97 (65.5)	42 (28.2)	
What do you think is the effectiveness of COVID-19 vaccines in protecting against the disease (what percentage of people vaccinated will not contract COVID 19)?				<0.001

about 20%	17 (5.7)	3 (2)	14 (9.5)	
about 40%	35 (11.8)	10 (6.7)	25 (16.9)	
about 70%	41 (13.8)	14 (9.4)	27 (18.2)	
above 90%	204 (68.7)	121 (81.9)	82 (55.4)	
Do you think that vaccination against COVID-19 is safe in pregnancy?				
yes	172 (57.5)	101 (67.3)	71 (47.6)	0.002
no	53 (17.7)	22 (14.7)	31 (20.8)	
depends on the trimester of pregnancy	43 (14.4)	12 (8)	31 (20.8)	
I do not know	31 (10.4)	15 (10)	16 (10.7)	
Do you think the complications after receiving the COVID-19 vaccine are:				<0.001
very rare	127 (42.5)	85 (56.7)	42 (28.2)	
rare	105 (35.1)	47 (31.3)	58 (38.9)	
do not know	13 (4.3)	3 (2)	10 (6.7)	
common	24 (8.03%)	6 (4)	18 (12.1)	
very common	30 (10)	9 (6)	21 (14.1)	

Do you think that the complications of having the COVID-19 vaccine in pregnancy, compared to non-pregnant women, are:				0.001
less dangerous	19 (6.3)	10 (6.7)	9 (6)	
equally dangerous	207 (69.2)	118 (78.7)	89 (59.7)	
more dangerous	52 (17.4)	13 (8.7)	39 (26.2)	
do not know	19 (7)	7 (2)	12 (8)	
Is the COVID-19 vaccine dangerous to the foetus?				0.002
yes	52 (17.4)	19 (12.7)	33 (22.1)	
no	169 (56.5)	102 (68)	67 (45)	
do not know	53 (17.7)	21 (14)	31 (21.5)	
insufficient research on this topic	25 (8.4)	8 (5.3)	17 (11.4)	
Have you been vaccinated against COVID-19?				<0.001
yes	16 (5.3)	11 (7.3)	5 (3.4)	
yes, before pregnancy	19 (6.3)	13 (8.7)	6 (4)	
yes, during pregnancy	10 (3.3)	6 (4)	4 (2.7)	
the first and second dose during pregnancy	9 (3)	6 (4)	3 (2)	
I postponed the first dose before pregnancy - full vaccination until later in pregnancy	23 (7.7)	16 (10.7)	7 (4.7)	

I postponed the first dose during pregnancy - full vaccination after pregnancy	33 (11)	28 (18.7)	5 (3.4)	
no	189 (63.2)	70 (46.7)	119 (79.9)	
In the previous question, the answer was 'no'. Reason for the negative approach:				
1) Fear of post-vaccination complications/adverse reactions	120 (40)	28 (18.7)	92 (61.3)	<0.001
2) Fear of damage to the foetus	160 (53.3)	73 (48.7)	87 (58)	0.1
3) Fear of complications for pregnancy such as pre-term delivery	74 (24.7)	18 (12)	56 (37.3)	<0.001
4) I believe that vaccinations are ineffective	23 (7.7)	4 (2.7)	19 (12.7)	0.001
5) I believe that disease is better than vaccination and provides more long-lasting immunity	4 (1.3)	1 (0.7)	3 (2)	0.62
6) I have not had such an opportunity yet due to limitations in the vaccination programme	8 (2.7)	1 (0.7)	7 (4.7)	0.07
7) Other reasons	1 (0.3)	0 (0)	1 (0.7)	1.0
Are you concerned about the COVID-19 vaccination in pregnancy?				<0.001
yes	49 (16.6)	19 (12.8)	30 (20.4)	
probably yes	54 (18.3)	19 (12.8)	35 (23.8)	
probably no	102 (34.6)	46 (31.1)	56 (38.1)	

definitely not	90 (30.5)	64 (43.2)	26 (17.7)	
Are you going to get vaccinated against COVID-19 when you are pregnant (if you have not yet been vaccinated)?				<0.001
yes	106 (35.4)	75 (50)	31 (20.8)	
probably yes	72 (24.1)	34 (22.7)	38 (25.5)	
do not know	43 (14.4)	14 (9.3)	29 (19.5)	
no	59 (19.7)	23 (15.3)	36 (24.2)	
definitely not	19 (6.3)	4 (2.7)	15 (10.1)	
The question concerns women who answered 'no'/'rather not'/'definitely not' to the previous question. Reason for the negative approach:				
1) Fear of post-vaccination complications/adverse reactions	101 (33.7)	37 (24.7)	64 (42.7)	0.001
2) Fear of damage to the foetus	78 (26)	22 (14.7)	56 (37.3)	<0.001
3) Fear of complications for pregnancy such as pre-term delivery	37 (12.3)	4 (2.7)	33 (22)	<0.001
4) I believe that vaccinations are ineffective	17 (5.7)	4 (2.7)	13 (8.7)	0.02
5) I believe that disease is better than vaccination and provides more long-lasting immunity	5 (1.7)	1 (0.7)	4 (2.7)	0.37
6) Other reasons	2 (0.7)	1 (0.7)	1 (0.7)	1.0

Have you vaccinated your children against COVID-19?				<0.001
yes	81 (27.3)	59 (39.3)	22 (15)	
no	76 (25.6)	27 (18)	49 (33.3)	
I have not had such an opportunity yet due to the age restrictions in the vaccination programme	108 (36.4)	53 (35.3)	55 (37.4)	
I don't have children yet	32 (10.8)	11 (7.3)	21 (14.3)	
The question concerns women who answered 'no' to the previous question. Reason for the negative approach:				
1) Risk of post-vaccination complications/undesirable effects	134 (44.7)	57 (38)	77 (51.3)	0.02
2) I believe that vaccinations are ineffective	50 (16.7)	10 (6.7)	40 (26.7)	<0.001
3) I believe that disease is better than vaccination and provides more long-lasting immunity	31 (10.3)	3 (2)	28 (18.7)	<0.001
4) Other reasons	15 (5)	4 (2.7)	11 (7.3)	0.06
Are you planning to vaccinate your children against COVID-19?				<0.001
yes	135 (45.9)	88 (59.1)	47 (32.4)	
probably yes	84 (28.6)	40 (26.8)	44 (30.3)	
do not know	46 (15.6)	15 (10.1)	31 (21.4)	
no	22 (7.5)	4 (2.7)	18 (12.4)	

definitely not	7 (2.4)	2 (1.3)	5 (3.4)	
The question concerns women who answered 'no'/'rather not'/'definitely not' to the previous question. Reason for the negative approach:				
1) Risk of post-vaccination complications/undesirable effects	73 (24.3)	26 (17.3)	47 (31.3)	0.004
2) I believe that vaccinations are ineffective	36 (12)	8 (5.33)	28 (18.7)	<0.001
3) I believe that disease is better than vaccination and provides more long-lasting immunity	17 (5.67)	2 (1.33)	15 (10)	0.001
4) Other reasons	11 (3.7)	2 (1.33)	9 (6)	0.03
Would you like your relatives to get vaccinated against COVID-19?				<0.001
yes	167 (56.6)	95 (63.3)	72 (49.7)	
yes, but only relatives at risk of severe disease	64 (21.7)	35 (23.3)	29 (20)	
do not know	43 (14.6)	14 (9.3)	29 (20)	
no	21 (7.1)	6 (4)	15 (10.3)	
Definitely not				

The question concerns women who answered 'no'/'rather not'/'definitely not' to the previous question. Reason for the negative approach:	57 (19)	29 (19.3)	28 (18.7)	0.88
1) Risk of post-vaccination complications/undesirable effects	17 (5.7)	5 (3.3)	12 (8)	0.08
2) I believe that vaccinations are ineffective	0 (0)	0 (0)	0 (0)	-
3) I believe that disease is better than vaccination and provides more long-lasting immunity	0 (0)	0 (0)	0 (0)	-
4) Other reasons	0 (0)	0 (0)	0 (0)	-

Table S4. Socioeconomic indices- acceptance before and after medical consultation.

	**Before physician consultation**				**After physician consultation**		
	**Total**	**Disagree**	**Agree**	**P value**	**Disagree**	Agree	P-value
**Place of residence:**							
Rural are	49 (16.3)	45 (19.1)	4 (6.1)	0.001	34 (17.9)	14 (12.7)	0.05
City < 50000 residents	78 (26)	68 (28.9)	10 (15.4)		60 (31.7)	18 (16.3)	
City < 250000 residents	137 (45.7)	95 (40.4)	42 (64.6)		69 (36.5)	68 (61.8)	
City ≥ 250000 residents	36 (12)	27 (11.5)	9 (13.8)		26 (13.7)	10 (9.1)	
Education							
Primary	37 (12.3%)	30 (12.7%)	7 (10.7%)	0.006	21 (11.1%)	16 (14.55%)	0.009

Vocational	53 (17.6%)	48 (20.4%)	5 (7.7%)		44 (23.2%)	9 (8.1%)	
Secondary	108 (36.0%)	88 (37.4%)	20 (30.7%)		76 (40.2%)	32 (29.1%)	
Higher	102 (34.0%)	69 (29.4%)	33 (50.7%)		48 (25.4%)	53 (48.1%)	
Prior births	178 (59.3%)	144 (61.2%)	34 (52.3%)	0.19	122 (64.5%)	56 (50.9%)	0.02
Number of prior births<br>n<br>Mean (±SD)<br>Median (Q1;Q3)	300<br>0.95 ± 0.96<br>1.00<br>(0.00;2.00)	235<br>1.00 ± 0.97<br>1.00<br>(0.00;2.00)	65<br>0.75 (±0.90)<br>0.00<br>(0.00;1.50)	0.06	189<br>1.08 (±0.99)<br>1.00<br>(0.00;2.00)	110<br>0.72 (±0.88)<br>0.00<br>(0.00;1.00)	0.001

Number of children<br><br>n<br><br>Mean(±SD)<br><br>Median (Q1;Q3)	300<br><br>0.93 (±0.96)<br><br>1.00<br><br>(0.00; 2.00)	235<br><br>0.99 (±0.98)<br><br>1.00<br><br>(0.00;2.00)	65<br><br>0.72 (±0.86)<br><br>0.00<br><br>(0.00;1.00)	0.05	189<br><br>1.07 (±1.01)<br><br>1.00<br><br>(0.00;2.00)	110<br><br>0.71 (±0.82)<br><br>0.00<br><br>(0.00;1.00)	0.003
Prior miscarriages<br><br>n<br><br>Mean (±SD)<br><br>Median (Q1;Q3)	300<br><br>0.74 ± 0.75<br><br>1.00<br><br>(0.00;1.00)	235<br><br>0.76 ± 0.75<br><br>1.00<br><br>(0.00;1.00)	65<br><br>0.68 ± 0.75<br><br>1.00<br><br>(0.00;1.00)	0.39	189<br><br>0.78 ± 0.75<br><br>1.00<br><br>(0.00;1.00)	110<br><br>0.68 ± 0.75<br><br>1.00<br><br>(0.00;1.00)	0.24
Currently in a relationship							0.093

Married	190 (63.3%)	156 (66.3%)	34 (52.3%)		128 (67.7%)	61 (55.4%)	
Partnership	94 (31.3%)	68 (28.9%)	26 (40.0%)		53 (28.0%)	41 (37.2%)	
No relationship	16 (5.3%)	11 (4.6%)	5 (7.6%)		8 (4.2%)	8 (7.2%)	
Trimester of pregnancy				0.818			0.367
First	46 (15.3)	37 (15.7)	9 (13.8%)		27 (14.3%)	19 (17.2%)	
Second	144 (48)	112 (47.6)	32 (49.2%)		90 (47.6%)	54 (49.1%)	
Third	110 (36.6)	86 (36.60)	24 (36.9)		72 (38.1%	37 (33.6%)	
Chronic diseases	140 (46.6)	104 (44.2)	36 (55.3)	0.11	85 (44.9%)	55 (50.0%)	0.40
Medications	137 (45.6)	101 (42.9)	36 (55.3)	0.07	81 (42.8%)	56 (50.9%)	0.18
Allergies	102 (34)	70 (29.7)	32 (49.2)	0.003	64 (33.8%)	38 (34.5%)	0.90
Has your doctor talked to you about the COVID-19 vaccination?	84 (28.1)	48 (20.5)	36 (55.4)	<0.001	39 (20.7%)	45 (40.9%)	0.001

Do you think that the course of COVID-19 disease in pregnancy, compared to non-pregnant women, is usually				<0.001			0.02
Milder	145 (48.6%)	134 (57.5%)	11 (16.9%)		102 (54.5%)	43 (39.1%)	
Comparable	61 (20.4%)	45 (19.3%)	16 (24.6%)		37 (19.8%)	23 (20.9%)	
More dangerous	61 (20.4%)	35 (15.0%)	26 (40.0%)		29 (15.5%)	32 (29.1%)	
Much more dangerous	31 (10.4%)	19 (8.1%)	12 (18.4%)		19 (10.1%)	12 (10.9%)	
Do you think that immunity obtained by disease lasts longer than immunity acquired by vaccination?				<0.001			<0.001
Definitely yes	110 (36.6%)	106 (45.1%)	4 (6.1%)		85 (44.9%)	25 (22.7%)	
Probably yes	87 (29.0%)	79 (33.6%)	8 (12.3%)		61 (32.2%)	25 (22.7%)	
Probably not	58 (19.3%)	32 (13.6%)	26 (40.0%)		28 (14.8%)	30 (27.2%)	

Definitely not	45 (15.0%)	18 (7.6%)	27 (41.5%)		15 (7.9%)	30 (27.2%)	
What do you think is the effectiveness of COVID-19 vaccines in protecting against the disease (what percentage of people vaccinated will not contract COVID-19)?				<0.001			<0.001
About 20%	77 (25.6%)	70 (29.7%)	7 (10.7%)		26 (13.7%)	52 (47.2%)	
About 40%	94 (31.3%)	91 (38.7%)	3 (4.6%)		99 (52.3%)	29 (26.3%)	
About 70%	67 (22.3%)	48 (20.4%)	19 (29.2%)		29 (15.3%)	18 (16.3%)	
Above 90%	62 (20.6%)	26 (11.0%)	36 (55.3%)		35 (18.5%)	11 (10.0%)	
Do you think that vaccination against COVID-19 is safe in pregnancy?				<0.001			<0.001
Yes	78 (26.0%)	33 (14.0%)	45 (69.2%)		26 (13.7%)	52 (47.2%)	
No	128 (42.6%)	120 (51.0%)	8 (12.3%)		99 (52.3%)	29 (26.3%)	

It depends on the trimester of pregnancy	47 (15.6%)	38 (16.1%)	9 (13.8%)		29 (15.3%)	18 (16.3%)	
I do not know	47 (15.67%)	44 (18.7%)	3 (4.6%)		35 (18.5%)	11 (10.0%)	
Do you think the complications after receiving the COVID-19 vaccine are:				<0.001			<0.001
Very rare	27 (9.00%)	11 (4.6%)	16 (24.6%)		11 (5.8%)	16 (14.5%)	
Rare	89 (29.6%)	48 (20.4%)	41 (63.0%)		37 (19.5%)	51 (46.3%)	
I do not know	22 (7.3%)	20 (8.5%)	2 (3.0%)		20 (10.5%)	2 (1.8%)	
Common	88 (29.3%)	86 (3.%)	2 (3.0%)		67 (35.4%)	21 (19.0%)	
Very common	74 (24.6%)	70 (29.7%)	4 (6.1%)		54 (28.5%)	20 (18.1%)	
Do you think that the complications of getting the COVID-19 vaccine in pregnancy compared to non-pregnant women are:				<0.001			<0.001

Less dangerous	29 (9.6%)	24 (10.2%)	5 (7.6%)		19 (10.0%)	10 (9.0%)	
Equally dangerous	83 (27.6%)	33 (14.0%)	50 (76.9%)		26 (13.7%)	57 (51.8%)	
More dangerous	151 (50.3%)	144 (61.2%)	7 (10.7%)		115 (60.8%)	36 (32.7%)	
I do not know	37 (12.3%)	34 (14.4%)	3 (4.6%)		29 (15.3%)	7 (6.3%)	
Is the COVID-19 vaccine dangerous to the foetus?				<0.001			<0.001
Yes	164 (54.6%)	142 (60.4%)	22 (33.8%)		108 (57.1%)	56 (50.9%)	
No	56 (18.6%)	30 (12.7%)	26 (40.0%)		24 (12.70%)	32 (29.0%)	
I do not know	60 (20.0%)	49 (20.8%)	11 (16.9%)		43 (22.75%)	17 (15.4%)	
Insufficient research on this topic	20 (6.6%)	14 (5.9%)	6 (9.2%)		14 (7.41%)	5 (4.5%)	
Have you been vaccinated against COVID-19?				<0.001			<0.001
Yes	11 (3.6%)	0 (0.0%)	11 (16.9%)		2 (1.0%)	9 (8.18%)	

Yes, before pregnancy	16 (5.3%)	0 (0.0%)	16 (24.6%)		2 (1.0%)	14 (12.7%)	
Yes, during pregnancy	5 (1.6%)	0 (0.0%)	5 (7.6%)		0 (0.0%)	5 (4.5%)	
The first and second dose during pregnancy	6 (2.0%)	0 (0.0%)	6 (9.2%)		0 (0.0%)	6 (5.4%)	
I postponed the first dose before pregnancy - full vaccination until later in pregnancy	12 (4.0%)	0 (0.0%)	12 (18.4%)		1 (0.5%)	11 (10.0%)	
I postponed the first dose during pregnancy - full vaccination after pregnancy	15 (5.0%)	0 (0.0%)	15 (23.0%)		2 (1.0%)	13 (11.8%)	
No	235 (78.3%)	235 (100.0%)	0 (0.0%)		182 (96.3%)	52 (47.2%)	
In the previous question, the answer was 'no'. Reason for the negative approach:							
1) Fear of post-vaccination complications/adverse reactions	164 (69.7%)	9 (13.8%)	173 (57.6%)	<0.001	133 (70.3%)	40 (36.3%)	<0.001
2) Fear of damage to the foetus	201 (67.0%)	189 (80.4%)	12 (18.4%)	<0.001	144 (76.1%)	56 (50.9%)	<0.001
3) Fear of complications for pregnancy such as pre-term delivery	103 (34.3%)	98 (41.7%)	5 (7.6%)	<0.001	83 (43.9%)	20 (18.1%)	<0.001

4) I believe that vaccinations are ineffective	55 (18.3%)	55 (18.3%)	2 (3.0%)	0.028	49 (25.93%)	6 (5.45%)	0.028
5) I believe that disease is better than vaccination and provides more long-lasting immunity	17 (7.2%)	0 (0.0%)	17 (5.6%)	0.473	15 (7.9%)	2 (1.8%)	0.545
6) I have not had such an opportunity yet due to limitations in the vaccination programme	12 (4.0%)	11 (4.6%)	1 (1.5%)	1.0000	9 (4.76%)	3 (2.7%)	0.3
7) Other reasons	3 (1.0%)	0 (0.0%)	3 (1.0%)	<0.001	3 (1.5%)	0 (0.0%)	<0.001
Are you concerned about the COVID-19 vaccination in pregnancy?				<0.001			<0.001
Yes	114 (38.2%)	110 (47.2%)	4 (6.1%)		95 (50.8%)	19 (17.2%)	
Probably yes	103 (34.5%)	97 (41.6%)	6 (9.2%)		72 (38.5%)	30 (27.2%)	
Probably no	60 (20.1%)	22 (9.4%)	38 (58.4%)		19 (10.1%)	41 (37.2%)	
Definitely not	21 (7.0%)	4 (1.7%)	17 (26.1%)		1 (0.5%)	20 (18.1%)	
Are you going to be vaccinated against COVID-19 when you are				<0.001			<0.001

pregnant (if you have not yet been vaccinated)?							
Yes	37 (12.3%)	9 (3.8%)	28 (43.0%)		8 (4.2%)	29 (26.3%)	
Probably yes	41 (13.6%)	16 (6.8%)	25 (38.4%)		13 (6.8%)	28 (25.4%)	
I do not know	45 (15.0%)	41 (17.4%)	4 (6.1%)		31 (16.4%)	14 (12.7%)	
No	140 (46.6%)	136 (57.8%)	4 (6.1%)		111 (58.7%)	28 (25.4%)	
Definitely not	37 (12.3%)	33 (14.0%)	4 (6.1%)		26 (13.7%)	11 (10.0%)	
The question concerns women who answered 'no'/'rather not'/'definitely not' to the previous question. Reason for the negative approach:							
1) Fear of post-vaccination complications/adverse reactions	189 (63.0%)	179 (76.1%)	10 (15.3%)	<0.001	141 (74.6%)	48 (43.6%)	<0.001
2) Fear of damage to the foetus	178 (59.3%)	170 (72.3%)	8 (12.3%)	<0.001	131 (69.3%)	47 (42.7%)	<0.001
3) Fear of complications for pregnancy such as pre-term delivery	110 (36.6%)	108 (45.9%)	2 (3.0%)	<0.001	88 (46.5%)	21 (19.0%)	<0.001

4) I believe that vaccinations are ineffective	178 (59.3%)	45 (19.1%)	1 (1.5%)	0.2134	42 (22.2%)	4 (3.6%)	0.493
5) I believe that disease is better than vaccination and provides more long-lasting immunity	9 (3.0%)	9 (3.8%)	0%(0.0)	1.000	7 (3.7%)	2 (1.8%)	1.000
6) Other reasons	1 (0.4%)	0 (0.0%)	1 (0.4%)	<0.001	1 (0.5%)	0 (0.0%)	<0.001
Have you vaccinated your children against COVID-19?				<0.001			<0.001
Yes	33 (11.0%)	11 (4.6%)	22 (33.8%)		8 (4.2%)	25 (22.7%)	
No	122 (40.6%)	112 (47.6%)	10 (15.3%)		86 (45.5%)	36 (32.7%)	
I have not had such an opportunity yet due to the age restrictions in the vaccination programme	116 (38.6%)	83 (35.3%)	33 (50.7%)		71 (37.5%)	45 (40.9%)	
I don't have children yet	29 (9.6%)	29 (12.3%)	0 (0.0%)		24 (12.7%)	4 (3.6%)	
The question concerns women who answered 'no' to the previous question. Reason for the negative approach:							

1) Risk of post-vaccination complications/undesirable effects	187 (62.3%)	19 (29.2%)	168 (71.4%)	<0.001	136 (71.9%)	51 (46.3%)	<0.001
2) I believe that vaccinations are ineffective	87 (29.0%)	82 (34.8%)	5 (7.6%)	<0.001	70 (37.0%)	17 (15.4%)	<0.001
3) I believe that disease is better than vaccination and provides more long-lasting immunity	65 (21.6%)	64 (27.2%)	1 (1.5%)	0.006	54 (28.5%)	11 (10.0%)	0.014
4) Other reasons	23 (9.7%)	23 (9.7%)	0 (0.0%)	<0.001	20 (10.5%)	3 (2.7%)	<0.001
Are you planning to vaccinate your children against COVID-19?				<0.001			<0.001
Yes	46 (15.3%)	11 (4.7%)	35 (53.8%)		7 (3.7%)	39 (35.4%)	
Probably yes	49 (16.3%)	29 (12.3%)	20 (30.7%)		26 (13.7%)	23 (20.9%)	
I do not know	82 (27.42%)	73 (31.2%)	9 (13.8%)		62 (32.80%)	20 (18.1%)	
No	106 (35.4%)	105 (44.8%)	1 (1.5%)		79 (41.8%)	27 (24.5%)	
Definitely not	16 (5.3%)	16 (6.8%)	0 (0.0%)		15 (7.9%)	1 (0.9%)	

The question concerns women who answered 'no'/'rather not'/'definitely not' to the previous question. Reason for the negative approach:							
1) Risk of post-vaccination complications/undesirable effects	184 (61.3%)	172 (73.1%)	12 (18.4%)	<0.001	134 (70.9%)	50 (45.4%)	<0.001
2) I believe that vaccinations are ineffective	94 (31.3%)	91 (38.7%)	3 (4.6%)	<0.001	73 (38.6%)	21 (19.0%)	<0.001
3) I believe that disease is better than vaccination and provides more long-lasting immunity	73 (24.3%)	71 (30.2%)	2 (3.0%)	0.139	133 (70.3%)	93 (84.5%)	0.029
4) Other reasons	28 (9.3%)	25 (10.6%)	3 (4.6%)	<0.001	23 (12.1%)	5 (4.5%)	<0.001
Would you like your relatives to get vaccinated against COVID-19?				<0.001			<0.001
Yes	64 (21.3%)	36 (55.3%)	28 (11.9%)		25 (13.2%)	38 (34.5%)	
Yes, but only relatives at risk of severe disease	66 (22.0%)	26 (40.0%)	40 (17.0%)		31 (16.4%)	35 (31.8%)	
I do not know	68 (22.6%)	2 (3.0%)	66 (28.0%)		54 (28.5%)	14 (12.7%)	

No	93 (31.0%)	1 (1.5%)	92 (39.1%)		72 (38.1%)	21 (19.0%)	
Definitely not	9 (3.0%)	0 (0.0%)	9 (3.8%)		7 (3.7%)	2 (1.8%)	
The question concerns women who answered 'no'/'rather not'/'definitely not' to the previous question. Reason for the negative approach:							
1) Risk of post-vaccination complications/undesirable effects	187 (62.3%)	177 (75.3%)	10 (15.38%)	<0.001	140 (74.0%)	47 (42.7%)	<0.001
2) I believe that vaccinations are ineffective	95 (31.6%)	90 (38.3%)	5 (7.6%)	<0.001	75 (39.6%)	20 (18.1%)	<0.001
3) I believe that disease is better than vaccination and provides more long-lasting immunity	56 (18.6%)	56 (23.8%)	0 (0.0%)	0.03	49 (25.9%)	7 (6.3%)	0.09
4) Other reasons	17 (5.67)	17 (7.2)	0 (0)	0.54	14 (7.4)	3 (2.7)	<0.001

Data are presented as mean ± SD and median (Q1;Q3) for continuous variables or counts (percentages) for categorical variables

Table S5. General characteristics and acceptance before and after medical consultation.

	Total	Before physician consultation			After physician consultation		
		Disagree	Agree	P value	Disagree	Agree	p
Age, years	25.2 ± 4.3	24.8 ± 4.3	26.5 ± 4.2	0.002	25.0 ± 4.2	25.6 ± 4.6	0.25
Arterial hypertension	20 (6.7)	13 (5.5)	7 (10.8)	0.16	11 (5.8)	9 (8.2)	0.43
Epilepsy	9 (3.0)	8 (3.4)	1 (1.5)	0.69	7 (3.7)	2 (1.8)	0.49
Diabetes mellitus	32 (10.7)	26 (11.0)	6 (9.2)	0.67	22 (11.6)	10 (9.1)	0.49
Heart defect	8 (2.7)	5 (2.1)	3 (4.6)	0.38	6 (3.2)	2 (1.8)	0.71
Bronchial asthma	14 (4.7)	9 (3.8)	5 (7.7)	0.19	7 (3.7)	7 (3.7)	0.29
Pancreatitis	10 (3.3)	6 (2.5)	4 (6.1)	0.23	5 (2.6)	5 (2.6)	0.51
Obesity	31 (10.3)	2 (3.1)	29 (12.3)	0.03	23 (12.2)	8 (7.3)	0.18
Psoriasis	11 (3.7)	8 (3.4)	3 (4.6)	1.0	7 (3.7)	4 (3.6)	1.0
Migraine	5 (1.7)	4 (1.7)	1 (1.5)	0.71	0 (0)	5 (1.7)	0.16
Thrombophilia	10 (3.3)	9 (3.8)	1 (1.5)	0.70	6 (3.2)	4 (3.6)	1.0
Celiac disease	8 (2.7)	5 (2.1)	3 (4.6)	0.38	3 (1.6)	5 (4.5)	0.15
Sclerosis multiplex	9 (3.0)	7 (2.9)	2 (3.0)	1.0	3 (1.5)	6 (5.4)	0.08
Allergy	17 (5.6)	15 (6.3)	2 (3.0)	0.54	12 (6.3)	5 (4.5)	0.52

Depression	9 (3.0)	8 (3.4)	1 (1.54)	0.69	7 (3.7)	2 (1.8)	0.49
Bipolar affective disease	5 (1.6)	4 (1.7)	1 (1.5)	1.0	2 (1.0)	3 (2.7)	0.36
Melanoma	2 (0.6)	1 (0.4)	1 (1.5)	0.39	1 (0.5)	1 (1.54)	0.39
Cerebral tumour	1 (0.3)	1 (0.4)	0 (0)	1.0	1 (0.5)	0 (0)	1.0
Hypothyreosis	27 (9.0)	17 (7.2)	10 (15.3)	0.04	12 (6.3)	15 (13.6)	0.03

Data are presented as mean ± SD and counts (percentages).

Table S6. Other questions-acceptance before and after medical consultation.

	Before physician consultation				After physician consultation		
	**Total**	**Disagree**	**Agree**	***P*-value**	**Disagree**	**Agree**	***P*-value**
Do you think that the course of COVID-19 disease in pregnancy, compared to non-pregnant women, is usually:				<0.001			0.01
milder	145 (48.6)	134 (57.5)	11 (16.9)		102 (54.5)	43 (39.1)	
comparable	61 (20.4)	45 (19.3)	16 (24.6)		37 (19.8)	23 (20.9)	
more dangerous	61 (20.4)	35 (15)	26 (40)		29 (15.5)	32 (29.1)	
much more dangerous	31 (10.4)	19 (8.1)	12 (18.4)		19 (10.1)	12 (10.9)	
Do you think that immunity obtained by disease lasts longer than immunity acquired by vaccination?				<0.001			0.01
definitely yes	110 (36.6)	106 (45.1)	4 (6.1)		85 (44.9)	25 (22.7)	
probably yes	87 (29)	79 (33.6)	8 (12.3)		61 (32.2)	25 (22.7)	

probably not	58 (19.3)	32 (13.6)	26 (40)		28 (14.8)	30 (27.2)	
definitely not	45 (15)	18 (7.6)	27 (41.5)		15 (7.9)	30 (27.2)	
What do you think is the effectiveness of COVID-19 vaccines in protecting against the disease (what percentage of people vaccinated will not contract COVID-19)?				<0.001			<0.001
about 20%	77 (25.6%)	70 (29.7%)	7 (10.7%)		26 (13.7%)	52 (47.2%)	
about 40%	94 (31.3%)	91 (38.7%)	3 (4.6%)		99 (52.3%)	29 (26.3%)	
about 70%	67 (22.3%)	48 (20.4%)	19 (29.2%)		29 (15.3%)	18 (16.3%)	
about 90%	62 (20.6%)	26 (11.0%)	36 (55.3%)		35 (18.5%)	11 (10.0%)	
Do you think vaccination against COVID-19 is safe in pregnancy?				<0.001			<0.001
yes	78 (26.0%)	33 (14.0%)	45 (69.2%)		26 (13.7%)	52 (47.2%)	
no	128 (42.6%)	120 (51.0%)	8 (12.3%)		99 (52.3%)	29 (26.3%)	
depends on the trimester of pregnancy	47 (15.6%)	38 (16.1%)	9 (13.8%)		29 (15.3%)	18 (16.3%)	
do not know	47 (15.67%)	44 (18.7)	3 (4.6%)		35 (18.5%)	11 (10.0%)	

Do you think complications after vaccination against COVID-19 are:				<0.001			<0.001
very rare	27 (9)	11 (4.6)	16 (24.6)		11 (5.8%)	16 (14.5%)	
rare	89 (29.6)	48 (20.4)	41 (63)		37 (19.5%)	51 (46.3%)	
do not know	22 (7.3)	20 (8.5)	2 (3)		20 (10.5%)	2 (1.8%)	
often	88 (29.3)	86 (36.6)	2 (3)		67 (35.4%)	21 (19.0%)	
very often	74 (24.6)	70 (29.7)	4 (6.1)		54 (28.5%)	20 (18.1%)	
Do you think that complications after vaccination against COVID-19 in pregnancy, compared to non-pregnant women, are				<0.001			<0.001
less dangerous	29 (9.6)	24 (10.2)	5 (7.6)		19 (10)	10 (9)	
equally dangerous	83 (27.6)	33 (14)	50 (76.9)		26 (13.7)	57 (51.8)	
,ore dangerous	151 (50.3)	144 (61.2)	7 (10.7)		115 (60.8)	36 (32.7)	
do not know	37 (12.3)	34 (14.4)	3 (4.6)		29 (15.3)	7 (6.3)	
Is the COVID-19 vaccine dangerous to the foetus?				<0.001			<0.001

yes	164 (54.6%)	142 (60.4%)	22 (33.8%)		108 (57.1)	56 (50.9)	
no	56 (18.6%)	30 (12.7%)	26 (40.0%)		24 (12.7)	32 (29)	
do not know	60 (20.0%)	49 (20.8%)	11 (16.9%)		43 (22.7)	17 (15.4)	
Insufficient research on this topic	20 (6.6%)	14 (5.9%)	6 (9.2%)		14 (7.4)	5 (4.5)	
Have you been vaccinated against COVID-19?				<0.001			<0.001
yes	11 (3.6)	0 (0)	11 (16.9)		2 (1)	9 (8.18)	
yes, before pregnancy	16 (5.3)	0 (0)	16 (24.6)		2 (1)	14 (12.7)	
yes, during pregnancy	5 (1.6)	0 (0)	5 (7.6)		0 (0)	5 (4.5)	
The first dose during pregnancy, the second dose during pregnancy	6 (2)	0 (0)	6 (9.2)		0 (0)	6 (5.4)	
I postponed the first dose before pregnancy - full vaccination until later in pregnancy	12 (4)	0 (0)	12 (18.4)		1 (0.5)	11 (10)	
I postponed the first dose during pregnancy - full vaccination after pregnancy	15 (5)	0 (0)	15 (23)		2 (1)	13 (11.8)	
no	235 (78.3%)	235 (100)	0 (0)		182 (96.3)	52 (47.2)	

The question concerns women who answered 'no' to the previous question. Reason for the negative approach:							
1) Fear of post-vaccination complications/adverse reactions	164 (69.7)	9 (13.8)	173 (57.6)	<0.001	133 (70.3)	40 (36.3)	<0.001
2) Fear of damage to the foetus	201 (67)	189 (80.4)	12 (18.4%)	<0.001	144 (76.1%)	56 (50.9%)	<0.001
3) Fear of complications for pregnancy such as pre-term labour	103 (34.3%)	98 (41.7)	5 (7.6%)	<0.001	83 (43.9%)	20 (18.1%)	<0.001
4) I believe that vaccinations are ineffective	55 (18.3%)	55 (18.3)	2 (3)	0.03	49 (25.93%)	6 (5.45%)	0.028
5) I believe that going through the disease is better than vaccination and provides more long-lasting immunity	17 (7.2%)	0 (0)	17 (5.6%)	0.473	15 (7.9%)	2 (1.8%)	0.545
6) I have not had such an opportunity yet due to the limitations in the vaccination programme	12 (4)	11 (4.6)	1 (1.5%)	1.0000	9 (4.8)	3 (2.7%)	0.300
7) Other reasons	3 (1)	0 (0)	3 (1)	<0.001	3 (1.5)	0 (0)	<0.001
Are you concerned about the COVID-19 vaccination during pregnancy?				<0.001			<0.001
yes	114 (38.2)	110 (47.2)	4 (6.1)		95 (50.8)	19 (17.2)	
probably yes	103 (34.5%)	97 (41.6%)	6 (9.2%)		72 (38.5%)	30 (27.2%)	

probably not	60 (20.1%)	22 (9.4%)	38 (58.4%)		19 (10.1%)	41 (37.2%)	
definitely not	21 (7.0%)	4 (1.7%)	17 (26.1%)		1 (0.5%)	20 (18.1%)	
Do you intend to vaccinate against COVID-19 during pregnancy if you have not yet been vaccinated)?				<0.001			<0.001
yes	37 (12.3%)	9 (3.8%)	28 (43.0%)		8 (4.2%)	29 (26.3%)	
Probably yes	41 (13.6%)	16 (6.8%)	25 (38.4%)		13 (6.8%)	28 (25.4%)	
do not know	45 (15.0%)	41 (17.4%)	4 (6.1%)		31 (16.4%)	14 (12.7%)	
no	140 (46.6%)	136 (57.8%)	4 (6.1%)		111 (58.7%)	28 (25.4%)	
definitely not	37 (12.3%)	33 (14.0%)	4 (6.1%)		26 (13.7%)	11 (10.0%)	
The question concerns women who answered 'no'/'rather not'/'definitely not' in the previous question. Reason for the negative approach:							
1) Fear of post-vaccination complications/adverse reactions	189 (63.0%)	179 (76.1%)	10 (15.3%)	<0.001	141 (74.6%)	48 (43.6%)	<0.001
2) Fear of damage to the foetus	178 (59.3%)	170 (72.3%)	8 (12.3%)	<0.001	131 (69.3%)	47 (42.7%)	<0.001
3) Fear of complications for pregnancy such as pre-term labour	110 (36.6%)	108 (45.9%)	2 (3.0%)	<0.001	88 (46.5%)	21 (19.0%)	<0.001

4) I believe that vaccinations are ineffective	178 (59.3%)	45 (19.1%)	1 (1.5%)	0.21	42 (22.2%)	4 (3.6%)	0.493
5) I believe that going through the disease is better than vaccination and provides more long-lasting immunity	9 (3.0%)	9 (3.8%)	0%(0.0)	1.000	7 (3.7%)	2 (1.8%)	1.000
6) Other reasons	1 (0.4%)	0 (0.0%)	1 (0.4%)	<0.001	1 (0.5%)	0 (0.0%)	<0.001
Have you vaccinated your children against COVID-19?				<0.001			<0.001
yes	33 (11.0%)	11 (4.6%)	22 (33.8%)		8 (4.2%)	25 (22.7%)	
no	122 (40.6%)	112 (47.6%)	10 (15.3%)		86 (45.5%)	36 (32.7%)	
I have not had such an opportunity yet due to the age restrictions in the vaccination programme	116 (38.6%)	83 (35.3%)	33 (50.7%)		71 (37.5%)	45 (40.9%)	
I don't have children yet	29 (9.6%)	29 (12.3%)	0 (0.0%)		24 (12.7%)	4 (3.6%)	
The question concerns women who answered 'no' to the previous question. Reason for the negative approach:							
1) Risk of complications/adverse reactions from vaccination	187 (62.3%)	19 (29.2%)	168 (71.4%)	<0.001	136 (71.9%)	51 (46.3%)	<0.001

2) I believe that vaccinations are ineffective	87 (29.0%)	82 (34.8%)	5 (7.6%)	<0.001	70 (37.0%)	17 (15.4%)	<0.001
3) I believe that going through the disease is better than vaccination and provides more long-lasting immunity	65 (21.6%)	64 (27.2%)	1 (1.5%)	0.006	54 (28.5%)	11 (10.0%)	0.014
4) Other reasons	23 (9.7%)	23 (9.7%)	0 (0.0%)	<0.001	20 (10.5%)	3 (2.7%)	<0.001
Have you vaccinated your children against COVID 19?				<0.001			<0.001
yes	46 (15.3)	11 (4.7%)	35 (53.8%)		7 (3.7%)	39 (35.4%)	
probably yes	49 (16.3)	29 (12.3%)	20 (30.7%)		26 (13.7%)	23 (20.9%)	
do not know	82 (27.4)	73 (31.2%)	9 (13.8%)		62 (32.80%)	20 (18.1%)	
no	106 (35.4)	105 (44.8%)	1 (1.5%)		79 (41.8%)	27 (24.5%)	
definitely not	16 (5.3)	16 (6.8%)	0 (0)		15 (7.9%)	1 (0.9%)	
The question concerns women who answered 'no'/'rather not'/'definitely not' in the previous question.. Reason for the negative approach:							
1) Risk of complications/adverse reactions from vaccination	184 (61.3%)	172 (73.1%)	12 (18.4%)	<0.001	134 (70.9%)	50 (45.4%)	<0.001
2) I believe that vaccinations are ineffective	94 (31.3%)	91 (38.7%)	3 (4.6%)	<0.001	73 (38.6%)	21 (19.0%)	<0.001

3) I believe that going through the disease is better than vaccination and provides more long-lasting immunity	73 (24.3%)	71 (30.2%)	2 (3)	0.14	133 (70.3%)	93 (84.5%)	0.029
4) Other reasons	28 (9.3%)	25 (10.6%)	3 (4.6%)	<0.001	23 (12.1%)	5 (4.5%)	<0.001
Would you like your relatives to get vaccinated against COVID-19?				<0.001			<0.001
yes	64 (21.3)	36 (55.3)	28 (11.9)		25 (13.2)	38 (34.5)	
yes, but only relatives at risk of developing severe disease	66 (22)	26 (40)	40 (17)		31 (16.4)	35 (31.8)	
do not know	68 (22.6)	2 (3)	66 (28)		54 (28.5)	14 (12.7)	
no	93 (31)	1 (1.5)	92 (39.1)		72 (38.1)	21 (19)	
definitely not	9 (3)	0 (0)	9 (3.8)		7 (3.7)	2 (1.8)	
The question concerns women answered 'no'/'rather not'/'definitely not' in the previous question.. Reason for the negative approach:							
1) Risk of complications/adverse reactions from vaccination	187 (62.3)	177 (75.3)	10 (15.4)	<0.001	140 (74)	47 (42.7)	<0.001
2) I believe that vaccinations are ineffective	95 (31.6)	90 (38.3)	5 (7.6)	<0.001	75 (39.6)	20 (18.1)	<0.001

3) I believe that going through the disease is better than vaccination and provides more long-lasting immunity	56 (18.6)	56 (23.8)	0 (0)	0.03	49 (25.9)	7 (6.3)	0.09
4) Other reasons	17 (5.67)	17 (7.2)	0 (0)	0.54	14 (7.4)	3 (2.7)	<0.001

Data are presented as counts (percentages)

Table S7. Predictors of vaccination against Covid-19 – univariate logistic regression before consultation with physicians.

**Variable**	**OR per**	**OR (95% CI)**	***P*-value**	**AUC**
Country	Ukraine/Poland	0.272 (0.144; 0.491)	<0.001	0.652
Age	1 year	1.093 (1.026; 1.165)	0.005	0.621
Age	5 years	1.558 (1.139; 2.144)	0.005	0.621
Obesity	yes vs. no	0.226 (0.036; 0.778)	0.01	0.546
Accommodation	1 unit	1.744 (1.257; 2.470)	<0.001	0.629
Education	1 unit	1.519 (1.129; 2.090)	0.005	0.617

Number of children	1 child	0.731 (0.530; 0.987)	0.04	0.573
Allergies	yes vs. no	2.286 (1.303; 4.015)	0.004	0.597
14. Do you think that the course of COVID-19 disease in pregnancy, compared to non-pregnant women, is usually: 1. milder 2. comparable 3. more dangerous 4. much more dangerous?	1 unit	2.135 (1.639; 2.817)	<0.001	0.728
15. Do you think that immunity obtained through disease lasts longer than that acquired through vaccination? 1.definitely yes 2.probably yes 3.probably not 4.definitely not.	1 unit	3.735 (2.707; 5.335)	<0.001	0.832
16. What do you think the effectiveness of COVID-19 vaccines is in protecting against the disease (what percentage of people vaccinated will not contract COVID-19)?	1 unit	3.244 (2.357; 4.610)	<0.001	0.792

1. About 20% 2. About 40% 3. About 70% 4. Above 90%				
18. Do you think that the complications following the administration of the COVID-19 vaccine are: 1. very rare 2. rare 3. I do not know/common 4. common 5. very common	1 unit	0.271 (0.181; 0.388)	<0.001	0.810
21a. The question concerns women who answered 'no' to the previous question. Reason for negative approach: 1) Fear of vaccine complications/adverse reactions.	yes vs. no	0.070 (0.031; 0.142)	<0.001	0.780
21a. The question concerns women who answered 'no' in the previous question. Reason for negative approach: 2) Fear of harm to the foetus.	yes vs. no	0.055 (0.026; 0.108)	<0.001	0.810
21a. The question concerns women who answered 'no' to the previous question. Reason for negative approach: 3) Fear of pregnancy complications such as pre-term delivery.	yes vs. no	0.116 (0.040; 0.275)	<0.001	0.670
21a. The question concerns women who answered 'no' to the previous	yes vs. no	0.109 (0.018; 0.365)	<0.001	0.597

question. Reason for negative attitude: 4) I believe that vaccinations are ineffective.				
22. Are you concerned about the COVID-19 vaccination during pregnancy?		8.329 (5.157; 14.534)	<0.001	0.884
23. Are you going to get vaccinated against COVID-19 during pregnancy if you have not yet been vaccinated?	1 unit	0.250 (0.178; 0.339)	<0.001	0.860
23a. The question concerns women who answered 'no'/'rather not'/'definitely not' to the previous question. Reason for negative approach: 1) Fear of vaccine-related complications/adverse reactions.	yes vs. no	0.057 (0.026; 0.114)	<0.001	0.804
23a. The question concerns women who answered 'no' 'rather not'/'definitely not' to the previous question. Reason for negative approach: 2) Fear of harm to the foetus.	yes vs. no	0.054 (0.023; 0.113)	<0.001	0.800
23a. The question concerns women who answered 'no'/ 'rather not'/'definitely not' tp the previous question. Reason for negative approach:	yes vs. no	0.037 (0.006; 0.123)	<0.001	0.714

3) Fear of pregnancy complications such as pre-term delivery.				
23a. The question concerns women who answered 'no'/'rather not'/'definitely not' to the previous question. Reason for negative attitude: 4) I believe that vaccinations are ineffective.	yes vs. no	0.066 (0.004; 0.312)	<0.001	0.588
24a. The question concerns women who answered 'no' to the previous question. Reason for negative approach: 1) Risk of vaccine-related complications/adverse reactions.	yes vs. no	0.165 (0.088; 0.297)	<0.001	0.711
24a. The question concerns women who answered 'no' to the previous question. Reason for negative attitude: 2) I believe that vaccinations are ineffective.	yes vs. no	0.155 (0.053; 0.368)	<0.001	0.636
24a. The question concerns women who answered 'no' to the previous question. Reason for negative approach: 3) I believe that going through the disease is better than vaccination and proves more long-lasting immunity.	yes vs. no	0.042 (0.002; 0.195)	<0.001	0.628

25. Will you vaccinate your child/children against COVID-19?	1 unit	0.173 (0.111; 0.254)	<0.001	0.898
25a. The question concerns women who answered 'no'/'rather not'/'definitely not' to the previous question. Reason for negative approach: 1) Risk of vaccine-related complications/adverse reactions.	yes vs. no	0.083 (0.040; 0.160)	<0.001	0.774
25a. The question concerns women who answered 'no' / 'rather not'/'definitely not' to the previous question. Reason for negative attitude: 2) I believe that vaccinations are ineffective.	yes vs. no	0.077 (0.018; 0.215)	<0.001	0.670
25a. The question concerns women who answered 'no'/'rather not'/'definitely not' to the previous question. Reason for negative approach: 3) I believe that going through the disease is better than vaccination and provides more long-lasting immunity.	yes vs. no	0.073 (0.012; 0.243)	<0.001	0.636
26. Would you like your relatives to get vaccinated against COVID-19?	1 unit	0.217 (0.142; 0.314)	<0.001	0.860
26a. The question concerns women who answered 'no'/'rather	yes vs. no	0.060 (0.027; 0.120)	<0.001	0.799

not'/'definitely not' to the previous question. Reason for negative approach: 1) Risk of vaccine-related complications/adverse reactions.				
26a. The question concerns women who answered 'no' / 'rather not'/'definitely not' to the previous question. Reason for negative attitude: 2) I believe that vaccinations are ineffective.	yes vs. no	0.134 (0.046; 0.317)	<0.001	0.653
2. Do you think that immunity obtained through disease lasts longer than that acquired through vaccination?	1 unit	2.316 (1.597; 3.541)	<0.001	0.678
3. What do you think the effectiveness of COVID-19 vaccines is in protecting against the disease (what percentage of people vaccinated will not contract COVID 19)?	1 unit	2.241 (1.454; 3.840)	<0.001	0.618
5. Do you think that complications after vaccination against COVID-19 are:	1 unit	0.372 (0.234; 0.552)	<0.001	0.716
8a. The question concerns women who answered 'no' to the previous	yes vs. no	0.049 (0.012; 0.136)	<0.001	0.726

question. Reason for negative approach: 1) Fear of vaccine-related complications/adverse reactions.				
8a. The question concerns women who answered 'no' to the previous question. Reason for negative approach: 2) Fear of harm to the foetus.	yes vs. no	0.118 (0.056; 0.229)	<0.001	0.732
8a. The question concerns women who answered 'no' to the previous question. Reason for negative approach: 3) Fear of pregnancy complications such as pre-term delivery.	yes vs. no	0.112 (0.027; 0.315)	<0.001	0.628
8a. The question concerns women who answered 'no' to the previous question. Reason for negative attitude: 4) I believe that vaccinations are ineffective.	yes vs. no	0.151 (0.008; 0.743)	0.01	0.539
9. Are you concerned about vaccination against COVID-19 during pregnancy?	1 unit	3.372 (2.284; 5.249)	<0.001	0.773
10. Are you going to get vaccinated against COVID-19 when you are	1 unit	0.451 (0.326; 0.601)	<0.001	0.738

pregnant if you have not yet been vaccinated?				
10a. The question concerns women who answered 'no'/ 'rather not'/'definitely not' to the previous question. Reason for negative approach: 1) Fear of vaccine-related complications/adverse reactions.	yes vs. no	0.288 (0.132; 0.570)	<0.001	0.617
10a. The question concerns women who answered 'no'/ 'rather not'/'definitely not' to the previous question. Reason for negative approach: 2) Fear of harm to the foetus.	yes vs. no	0.143 (0.042; 0.363)	<0.001	0.627
10a. The question concerns women who answered 'no'/'rather not'/'definitely not' to the previous question. Reason for negative approach: 3) Fear of pregnancy complications such as pre-term delivery.	yes vs. no	0.086 (0.005; 0.412)	<0.001	0.569
11a. The question concerns women who answered 'no' to the previous question. Reason for negative approach: 1) Risk of vaccine-related complications/adverse reactions.	yes vs. no	0.292 (0.151; 0.538)	<0.001	0.638

11a. The question concerns women who answered 'no' to the previous question. Reason for negative approach: 2) I believe that vaccinations are ineffective.	yes vs. no	0.194 (0.046; 0.553)	0.001	0.577
12. Will you vaccinate your child/children against COVID-19?	1 unit	0.295 (0.175; 0.459)	<0.001	0.733
12a. The question concerns women who answered 'no'/ 'rather not'/'definitely not' in the previous question. Reason for negative approach: 1) Risk of vaccine-related complications/adverse reactions.	yes vs. no	0.367 (0.155; 0.772)	0.007	0.577
13. Would you like your relatives to be vaccinated against COVID-19?	1 unit	0.287 (0.156; 0.469)	<0.001	0.694
13a. The question concerns women who answered 'no'/ 'rather not'/'definitely not' to the previous question. Reason for negative approach: 1) Risk of vaccine-related complications/adverse reactions.	yes vs. no	0.225 (0.066; 0.579)	0.001	0.582

Table S8. Predictors of vaccination against COVID-19 – univariate logistic regression after consultation with physician.

**Variable**	**OR per**	**OR (95% CI)**	***P*-value**	**AUC**
Country	Ukraine/Poland	0.221 (0.131; 0.365)	<0.001	0.678
Hypothyroidism	yes vs. no	2.329 (1.050; 5.272)	0.04	0.536
2. Place of residence	1 unit	1.306 (1.002; 1.715)	0.04	0.572
3. Education	1 unit	1.381 (1.083; 1.779)	0.009	0.606
4. Prior births	yes vs. no	0.570 (0.352; 0.918)	0.02	0.568
5. Number of prior births	1 unit	0.654 (0.498; 0.848)	0.001	0.606
6. Number of children	1 unit	0.658 (0.502; 0.853)	0.001	0.595
14. Do you think that the course of COVID-19 disease in pregnancy, compared to non-pregnant women, is usually:	1 unit	1.314 (1.052; 1.646)	0.016	0.584
15. Do you think that immunity obtained by disease lasts longer than	1 unit	1.939 (1.538; 2.471)	<0.001	0.683

immunity acquired by vaccination?				
16. What do you think is the effectiveness of COVID-19 vaccines in protecting against the disease (what percentage of people vaccinated will not contract COVID 19)?	1 unit	1.988 (1.571; 2.544)	<0.001	0.693
18. Do you think that complications after vaccination against COVID 19 are:	1 unit	0.514 (0.398; 0.654)	<0.001	0.687
21a. The question concerns women who answered 'no' to the previous question. Reason for negative approach: 1) Fear of vaccine complications/adverse reactions.	yes vs. no	0.241 (0.145; 0.394)	<0.001	0.67
21a. The question concerns women who answered 'no' to the previous question. Reason for negative approach: 2) Fear of harm to the foetus.	yes vs. no	0.324 (0.195; 0.533)	<0.001	0.626
21a. The question concerns women who answered 'no' to the previous question. Reason for negative approach: 3) Fear of pregnancy complications such as pre-term delivery.	yes vs. no	0.284 (0.158; 0.491)	<0.001	0.629

21a. The question concerns women who answered 'no' to the previous question. Reason for negative approach: 4) ‘I believe that vaccinations are ineffective’.	yes vs. no	0.165 (0.061; 0.371)	<0.001	0.602
21a. The question concerns women who answered 'no' to the previous question. Reason for negative approach: 5) ‘I believe that going through the disease is better than vaccination and provides more long-term immunity’.	yes vs. no	0.215 (0.033; 0.781)	0.017	0.531
22. Are you concerned about the COVID-19 vaccination during pregnancy?	1 unit	3.639 (2.646; 5.151)	<0.001	0.7681
23. Are you going to be vaccinated against COVID-19 when you are pregnant (if you have not yet been vaccinated)?	1 unit	0.473 (0.376; 0.587)	<0.001	0.7124
23a. The question concerns women who answered 'no'/'rather not'/'definitely not' to the previous question. Reason for negative approach: 1) Fear of vaccine complications/adverse reactions.	yes vs. no	0.264 (0.159; 0.432)	<0.001	0.6548
23a. The question concerns women who answered 'no'/'rather not'/'definitely	yes vs. no	0.330 (0.202; 0.536)	<0.001	0.6329

not' to the previous question. Reason for negative approach: 2) Fear of harm to the foetus.				
23a. The question concerns women who answered 'no'/'rather not'/'definitely not' to the previous question. Reason for negative approach: 3) Fear of complications for pregnancy such as pre-term delivery.	yes vs. no	0.271 (0.153; 0.465)	<0.001	0.637
23a. The question concerns women who answered 'no'/'rather not'/'definitely not' to the previous question. Reason for negative approach: 4) ‘I believe that vaccinations are ineffective’.	yes vs. no	0.132 (0.039; 0.339)	<0.001	0.596
24a. The question concerns women who answered 'no' to the previous question. Reason for negative approach: 1) Risk of vaccine-related complications/adverse reactions.	yes vs. no	0.337 (0.205; 0.549)	<0.001	0.628
24a. The question concerns women who answered 'no' to the previous question. Reason for negative approach: 2) ‘I believe that vaccinations are	yes vs. no	0.311 (0.167; 0.553)	<0.001	0.608

ineffective'.				
24a. The question concerns women who answered 'no' to the previous question. Reason for negative approach: 3) 'I believe that going through the disease is better than vaccination and provides more long-lasting immunity'.	yes vs. no	0.278 (0.132; 0.540)	<0.001	0.5929
24a. The question concerns women who answered 'no' to the previous question. Reason for negative approach: 4) Other reasons.	yes vs. no	0.237 (0.055; 0.712)	0.008	0.5393
25. Will you vaccinate your child/children against COVID-19?	1 unit	0.434 (0.339; 0.547)	<0.001	0.7263
25a. The question concerns women who answered 'no'/'rather not'/'definitely not' to the previous question. Reason for negative approach: 1) Risk of vaccine-related complications/adverse reactions.	yes vs. no	0.342 (0.209; 0.556)	<0.001	0.627
25a. The question concerns women who 'no'/'rather not'/'definitely not' to the previous question. Reason for negative approach: 2) I believe that vaccinations are ineffective	yes vs. no	0.375 (0.211; 0.646)	<0.001	0.598

25a. The question concerns women who answered 'no'/'rather not'/'definitely not' to the previous question. Reason for negative approach: 3) ‘I believe that going through the disease is better than vaccination and provides more long-lasting immunity’.	yes vs. no	0.434 (0.232; 0.780)	0.005	0.571
25a. The question concerns women who answered 'no'/'rather not'/'definitely not' to the previous question. Reason for negative approach: 4) Other reasons.	yes vs. no	0.344 (0.113; 0.864)	0.02	0.538
26. Would you like your relatives to be vaccinated against COVID-19?	1 unit	0.546 (0.437; 0.675)	<0.001	0.688
26a. The question concerns women who answered 'no'/'rather not'/'definitely not' to the previous question. Reason for negative approach: 1) Risk of vaccine-related complications/adverse reactions.	yes vs. no	0.261 (0.158; 0.428)	<0.001	0.657
26a. The question concerns women who answered 'no'/'rather not /'definitely not' in the previous question. Reason for negative approach: 2) ‘I believe that vaccinations are ineffective’.	yes vs. no	0.338 (0.188; 0.585)	<0.001	0.607

26a. The question concerns women who answered 'no'/'rather not'/'definitely not' to the previous question. Reason for negative approach: 3) ‘I believe that going through the disease is better than vaccination and gives more long-lasting immunity’.	yes vs. no	0.194 (0.078; 0.420)	<0.001	0.598
1. Do you think that the course of COVID-19 disease in pregnancy, compared to non-pregnant women, is usually worse?	1 unit	1.639 (1.234; 2.210)	<0.001	0.629
2. Do you think that immunity obtained by disease lasts longer than immunity acquired by vaccination?	1 unit	2.877 (2.082; 4.116)	<0.001	0.727
3. What do you think the effectiveness of COVID-19 vaccines is in protecting against the disease (what percentage of people vaccinated will not contract COVID-19)?	1 unit	1.966 (1.431; 2.819)	<0.001	0.630
5. Do you think that complications after vaccination against COVID-19 are:	1 unit	0.269 (0.176; 0.390)	<0.001	0.755
8a. The question concerns women who answered 'no' to the previous	yes vs. no	0.031 (0.010; 0.072)	<0.001	0.7815

question. Reason for negative approach: 1) Fear of vaccine complications/adverse reactions.				
8a. The question concerns women who answered 'no' to the previous question. Reason for negative approach: 2) Fear of harm to the foetus.	yes vs. no	0.026 (0.012; 0.052)	<0.001	0.85
8a. The question concerns women who answered 'no' to the previous question. Reason for negative approach: 3) Fear of pregnancy complications such as pre-term delivery.	yes vs. no	0.146 (0.062; 0.302)	<0.001	0.64
8a. The question concerns women who answered 'no' to the previous question. Reason for negative approach: 4) I believe that vaccinations are ineffective.	yes vs. no	0.070 (0.004; 0.339)	<0.001	0.55
9. Are you concerned about the COVID-19 vaccination during pregnancy?	1 unit	3.474 (2.520; 4.961)	<0.001	0.78
10. Do you intend to get vaccinated against COVID-19 during pregnancy (if you have not yet been vaccinated)?	1 unit	0.369 (0.277; 0.476)	<0.001	0.78

10a. The question concerns women who answered 'no'/'rather not'/'definitely not' to the previous question. Reason for negative approach: 1) Fear of vaccine complications/adverse reactions.	yes vs. no	0.171 (0.088; 0.312)	<0.001	0.67
10a. The question concerns women who answered 'no'/'rather not'/'definitely not' to the previous question. Reason for negative approach: 2) Fear of harm to the foetus.	yes vs. no	0.155 (0.069; 0.311)	<0.001	0.64
10a. The question concerns women who answered 'no'/'rather not'/'definitely not' to the previous question. Reason for negative approach: 3) Fear of pregnancy complications such as pre-term delivery.	yes vs. no	0.128 (0.030; 0.367)	<0.001	0.58
11a. The question concerns women who answered 'no' to the previous question. Reason for negative approach: 1) Risk of vaccine-related complications/adverse reactions.	yes vs. no	0.286 (0.170; 0.474)	<0.001	0.65
11a. The question concerns women who answered 'no' to the previous	yes vs. no	0.231 (0.092; 0.503)	<0.001	0.582

question. Reason for negative approach: 2) I believe that vaccinations are ineffective.				
11a. The question concerns women who answered 'no' to the previous question. Reason for negative approach: 3) I believe that going through the disease is better than vaccination and gives more long-lasting immunity.	yes vs. no	0.049 (0.003; 0.232)	<0.001	0.57
12. Will you vaccinate your child/children against COVID-19?	1 unit	0.279 (0.186; 0.398)	<0.001	0.75
12a. The question concerns women who answered 'no'/'rather not'/'definitely not' to the previous question. Reason for negative approach: 1) Risk of vaccine-related complications/adverse reactions.	yes vs. no	0.321 (0.164; 0.594)	0.002	0.59
12a. The question concerns women who answered 'no'/'rather not'/'definitely not' to the previous question. Reason for negative approach: 2) I believe that vaccinations are ineffective.	yes vs. no	0.040 (0.002; 0.191)	<0.001	0.5880
13. Would you like your relatives to be vaccinated against COVID-19?	1 unit	0.260 (0.164; 0.387)	<0.001	0.72

13a. The question concerns women who answered 'no'/'rather not'/'definitely not' to the previous question. Reason for negative approach: 1) Risk of vaccine-related complications/adverse reactions.	yes vs. no	0.302 (0.138; 0.603)	<0.001	0.58
